# Advents in the Diagnosis and Management of Ischemic Colitis

**DOI:** 10.3389/fsurg.2017.00047

**Published:** 2017-09-04

**Authors:** Evangelos P. Misiakos, Dimitrios Tsapralis, Theodore Karatzas, Irene Lidoriki, Dimitrios Schizas, George S. Sfyroeras, Konstantinos G. Moulakakis, Chrysostomos Konstantos, Anastasios Machairas

**Affiliations:** ^1^3rd Department of Surgery, School of Medicine, National and Kapodistrian University of Athens, Attikon University Hospital, Athens, Greece; ^2^Department of General Surgery, General Hospital/Health Center of Ierapetra, Ierapetra, Greece; ^3^2nd Department of Propedeutic Surgery, School of Medicine, National and Kapodistrian University of Athens, Laiko Hospital, Athens, Greece; ^4^1st Department of Surgery, School of Medicine, National and Kapodistrian University of Athens, Laiko Hospital, Athens, Greece; ^5^Department of Vascular Surgery, School of Medicine, National and Kapodistrian University of Athens, Attikon University Hospital, Athens, Greece; ^6^2nd Department of Radiology, School of Medicine, National and Kapodistrian University of Athens, Attikon University Hospital, Athens, Greece

**Keywords:** ischemia, colon, necrosis, perforation, sepsis

## Abstract

**Background:**

Ischemic colitis (IC) is a common type of ischemic insult, resulting from decreased arterial blood flow to the colon. This disease can be caused from either atherosclerotic occlusive vascular disease or non-occlusive disease. The aim of this study is to present the diagnostic methodology and management of this severe disease based on current literature.

**Methods:**

A literature search has been done including articles referring to modern diagnosis and management of IC.

**Results:**

IC is usually a transient disease, but it can also cause gangrene of the colon, requiring emergency surgical exploration. Diagnosis is troublesome and is based on imaging examinations, mainly computerized tomography, which in association with colonoscopy can delineate the distribution pattern and severity of disease.

**Conclusion:**

The majority of patients with mild disease have usually complete clinical recovery within a short period. The severe forms of the disease carry high morbidity and mortality rates and prompt surgical intervention is the only way to improve the associated severe prognosis.

## Introduction

Ischemic colitis (IC) is the most usual type of intestinal ischemia. It is an insult to the colonic wall resulting from diminished blood flow. This can range from superficial injury of the mucosa and submucosal layer to full thickness necrosis of the colonic wall (1). Most attacks are transient and resolve spontaneously, whereas others may result to gangrene and necrosis of the colon with resultant perforation and feculent peritonitis. It mostly occurs in old patients (80%) and mainly occurs among debilitated elderly women; its clinical presentation includes abdominal pain, diarrhea and rectal bleeding (2, 3). Upon suspicion, the patient requires admission to hospital for physiological support and risk stratification. It can usually be managed expectantly; however, in the severe form, emergency surgical treatment may be required (4).

This clinical entity has first been recognized in 1963 by Boley et al. (5) and has been further studied by Marston et al. in 1966 (6). However, the new clinical entity has not been the subject of comprehensive studies until 1992, when Brandt and Boley analyzed the clinical patterns of its presentation, which remained almost the same up to present (7). Yadav et al. published the results of a retrospective population-based cohort study in 445 patients who were diagnosed with IC from 1976 through 2009 (8). They found that the incidence of IC increased almost fourfold from 6.1 cases/100,000 person-years in the period 1976–1980 to 22.9/100,000 in 2005–2009. This was attributed mainly to the increasing life expectancy for patients with underline co-morbidities, especially atherosclerotic diseases (9).

There is a variety of factors predisposing to this condition, and the pathophysiology of this entity is very complex. In this review, we emphasize on the etiology, risk factors, clinical presentation, pathophysiology, diagnostic methodology, and treatment of this severe condition.

## Etiology and Anatomic Considerations

In most cases, IC represents a non-occlusive ischemia of the wall of the large bowel caused by a sudden decrease in blood flow in the small arterioles of the colon, resulting from a low-volume state (10, 11). Atherosclerotic disease, aortic surgery, and conditions causing transient hypotension have been considered as etiological factors, although in many cases, etiology may remain obscure. Other factors associated with the disease include the use of oral contraceptives, hereditary coagulopathies, cocaine abuse, strenuous physical activity, and bacterial pathogens, such as cytomegalovirus and *Escherichia coli* (12). Moreover, conditions such as constipation, hyperuricemia, and the smoking habit may contribute to the development of the disease especially in young adults (13). In general, the colon is more susceptible to ischemia, because it has an inherently lower blood flow than that of the small bowel and in contrast to the latter, its functional motor activity is followed by a drop in the blood flow (14).

There are two types of IC regarding the anatomic distribution of the disease:

– The left-sided IC, which presents with acute abdominal pain and lower gastrointestinal bleeding, is usually associated with low-flow states, coagulopathies, cardiac disease, and surgery of the abdominal aorta.
– The right-sided IC, which presents with severe abdominal pain but rarely bloody diarrhea, is usually associated with superior mesenteric artery stenosis or occlusion. It often occurs in patients with chronic renal failure requiring hemodialysis (15, 16). It may also be seen in patients with severe chronic heart disease, especially aortic stenosis.

The marginal artery of Drummond protects the colon from ischemia by forming a system of arcades connecting the major arteries and the meandering mesenteric artery (arc of Riolan) (1). Anatomically, two areas of the colon are considered more vulnerable for ischemia:

– The first vulnerable area is the splenic flexure (Griffith’s point). In this area, the marginal artery of Drummond is often tenuous and is absent in almost 5% of patients.
– The second vulnerable area is the rectosigmoid junction (Sudek’s point). This point lies distally to the last collateral connection with the sigmoid arteries (15). These two points have limited connections with the main arteries and, therefore, are vulnerable to low-flow states. Colon ischemia occurs in these two vulnerable areas in most cases.

The vasa recta (end-vessels providing blood to the wall of the colon) are less developed in the right colon in comparison to the left colon. Moreover, the vasa recta are very sensitive to vasospasm, and there is no sufficient collateral blood flow at this level. For the above reasons, the right colon is prone to ischemia from low-flow states (17).

The pathologic spectrum of IC included transient ischemia, chronic ischemia, and gangrene. If the ischemia is limited to the mucosa, which is the most vulnerable layer of the colon, the disease may be transient and recovery may be complete. More intense ischemia involving the muscularis may result in scarring and possibly to a chronic stricture. Ischemia involving the full thickness of the bowel wall may lead to gangrene and perforation of the colonic wall and finally fecal peritonitis and sepsis.

## Clinical Presentation

The classic symptomatology of IC consists of abdominal pain, hematochezia, and leucocytosis in an elderly patient (1). These symptoms vary considerably and depend mainly on the degree of the ischemia and the length and the thickness of the colon that is affected. Ischemia limited to a small segment of the mucosa may cause crampy abdominal pain, urgent desire to defecate, and minimal rectal bleeding. Abdominal tenderness usually extends to the distribution of the segment involved. An episode of transient hypoperfusion usually precedes the episode of pain. Bright red bleeding usually occurs when the left colon is involved, whereas maroon loose stool typically occurs when the right colon is affected. More severe and extensive intestinal ischemia may result in acute abdominal pain and rebound tenderness over the diseased part of the large bowel, bacterial translocation, fever, leukocytosis, and acidosis.

Full thickness insult of the colonic wall as a result of severe compromise of its blood supply may result in colon perforation with resultant peritonitis and sepsis. More severe forms of the disease usually follow vascular operations, such as abdominal aortic aneurysm repair (18).

The clinical patterns of IC are still based on the classification of Brandt and Boley and depend largely on the degree of the histopathological damage in the colonic wall: (1) reversible colopathy (submucosal or intramural bleeding), (2) transient colitis, (3) chronic segmental ischemia, (4) gangrenous colitis, and (5) universal fulminant colitis (7, 19).

The non-gangrenous type accounts for almost 80–85% of cases. In this form, the disease is transient and usually reversible (20). Chronic forms usually present as chronic segmental colitis and may result in stricture formation. Gangrene occurs in 15% of cases, is accompanied by perforation and sepsis, and requires immediate laparotomy. Fulminant pancolitis is rare, occurs in 1% of the total cases, and may be lethal (20).

## Risk Factors

Noh et al. (21) from Korea, in a retrospective study involving 50 patients who were treated surgically for IC, found that 78% of patients had co morbid conditions, such as cancer, hypertension, coronary artery disease, diabetes mellitus, nephropathy, previous abdominal or vascular surgery, respiratory disease, and liver cirrhosis. Hypertension was the most common co morbid condition (46%), followed by diabetes mellitus, nephropathy, and coronary artery disease. History of previous surgery, i.e., gastrointestinal surgery, abdominal aortic aneurysm surgery, or major cardiovascular surgery, was present in 48% of patients.

In another case control study, the presence of IC was associated with risk factors, such as age greater than 60 years, hypertension, diabetes mellitus, hemodialysis, hypoalbuminemia, and the use of constipation-inducing medications (22). Systemic lupus erythematosous, sickle cell crisis, and acute pancreatitis are also conditions associated with IC.

A group from Kaiser Permanente reviewed a series of 424 cases of IC and found that 400 (94%) cases took place in outpatient basis and only 6% took place in hospitalized patients (23). Constipation, irritable bowel syndrome, vasculitis, cocaine and methamphetamine use, endurance running, contraceptive pill use, smoking, hyperuricemia, and polymorphisms in the coagulation factor V and plasminogen activator inhibitor genes are other predisposing factors for IC in young adults (13, 24–28).

### Abdominal Aortic Aneurysm Repair

Ischemic colitis is a severe complication after abdominal aortic aneurysm repair (AAA), with a prevalence rate of 2.9% (18). This is a highly lethal complication, with an associated mortality rate exceeding 50% (29). It is probably due to interruption of a vital blood supply, such as the inferior mesenteric artery, during abdominal aortic reconstruction.

Perry et al. identified 89,967 patients undergoing surgery for AAA over a period of 2 years, based on a Nationwide Inpatient Sample database, in the US. The overall incidence of IC was 2.2%, and it was higher (8.9%) for repair of a ruptured aneurysm. It was also higher for open repair (1.9%) than endovascular repair (0.5%). Regardless of the surgical approach, IC was associated with increased morbidity and a two- to fourfold increase in mortality (1, 30).

Moghadamyeghanch et al. identified the following six perioperative factors associated with the development of IC: renal failure, suprarenal aneurysm extension, ruptured aneurysm before surgery, diabetes, bleeding disorder, and intraoperative and postoperative transfusions (Table 1) (18). Indeed in ruptured aneurysms, interruption of IMA blood flow and the need for transfusion may predispose to the development of IC (31). In addition, raised intraabdominal pressure is another important mechanism behind colonic hypoperfusion after ruptured AAA repair (32). As protective measures, reimplantation of the IMA during open repair and revascularization of the hypogastric artery in patients with ruptured aneurysms requiring transfusions may reduce the risk of IC after surgery (18). In this series, surgery was required in almost half of patients (49.3%) who developed IC postoperatively and the need for subsequent surgical treatment increased the mortality of patients by more than seven times (18).

**Table 1:** Recent (2014–2017) multicenter studies presenting outcomes of treatment of ischemic colitis (IC).

Reference	Year of publication	Type of study, country	Outcomes
Yngvadottir et al. (2)	2017	Retrospective National study Iceland	89 patients, 88% left colon, 11% required surgery, 30-day mortality rate 6%, 3-year recurrence rate 15%
Choi et al. (4)	2015	Retrospective, six centers, Korea	292 patients, group of severe IC had higher ratio of chronic kidney disease and higher ratio of involvement of the right colon
Moghadamyeghanch et al. (18)	2016	US database, retrospective cohort study	75 patients, predictive factors for IC: transfusions, rupture of aneurysm, proximal extension of the aneurysm, diabetes, renal failure under dialysis, and female gender. No significant difference between open repair and EVAR in the development of IC
Noh et al. (21)	2015	Retrospective study, Korea	50 patients underwent surgery for IC, mortality 30%, preceding cardiovascular surgery, surgical delay ≥3 days were independent risk factors for mortality
Arif et al. (33)	2016	Prospective study, Germany	224 patients operated on for IC after cardiac surgery. A total of 71% of these suffered low output syndrome. Elevated lactate values are significant predictor for colectomy
Sherid et al. (34)	2014	Multicenter retrospective study	118 patients, IC recurred in 8.5% of patients. Current smoking status and presence of AAA were risk factors for recurrence
Sherid et al. (35)	2014	Retrospective study, USA	CT angiogram in 34 patients, contrast-enhanced CT scan in 54 patients. Neither the need for surgery nor all cause mortality was different between the two groups. CT angiogram did not help to improve prognosis of IC
Kwak et al. (36)	2017	Retrospective study, Korea	75 patients with IC, 19 with gangrenous type, factors predictive for gangrenous colitis: absence of diarrhea and hematochezia, abdominal tenderness, and hypoalbuminemia
Sadler et al. (37)	2014	A population-based US Study	10,111 patients who underwent colectomy for IC, between 1993 and 2008. Mortality rate was 21.0% and significantly decreased at an annual rate of 4.5%. Mortality rate was associated with older age and co-morbidities, such as liver disease, renal disease, and heart failure

### Cardiac Surgery

Ischemic colitis after cardiovascular surgery with cardiopulmonary bypass (CPB) is a rare complication with an incidence of <1% and poor outcome with a mortality rate of 30–100% (33, 38). Distended or acute abdomen accompanied by low output syndrome with/without lower gastrointestinal tract bleeding may urge for direct diagnostic evaluation and prompt surgical intervention. CPB may cause systemic inflammatory response syndrome (SIRS) leading to barrier loss at the colonic mucosa. Moreover, regional differences in perfusion in the bowel wall, accompanied by hypothermia and normothermia and the use of vasoconstrictors or vasopressin may aggravate mucosal ischemia (33, 39). Duration of cross clamp, use of intra-aortic balloon pump support, and serum lactate concentrations >5 mmol/L were found to be significant predictive factors for the development of non-occlusive mesenteric ischemia after cardiac surgery (40, 41) Low cardiac output may lead to diminished splanchnic perfusion leading to irreversible damage to the intestinal wall. Elevated serum lactate levels associated with metabolic acidosis have been considered as the early indicator of mesenteric ischemia, although this has been debated (40, 42).

### Constipation

During that condition, increased intraluminal pressure reduces blood flow to the colonic mucosa, predisposing the patient to regional ischemia of the colonic wall (1, 43).

Kimura et al. in their study from Japan showed that the prevalence of constipation in young IC patients was higher than in young healthy adults, although this difference was not statistically significant (13). Similarly, using a research database, Suh et al. showed that the relative risk for IC was 2.78 times higher for patients with constipation (44). Use of laxatives is not indicated as they may worsen the problem, leading often to perforation of the colon.

### Coagulopathy

Thrombophilia seems to predispose to colonic ischemia, and abnormal clotting tests have been reported to be present from 28 to 74% of patients with IC (45). However, its role in the development of IC has not been clarified yet. In addition, antiphospholipid antibodies and factor V Leiden mutations are present 10 times more frequently in patients with IC (46). Moreover, conditions such as anti-thrombin deficiency, polycythemia vera, protein C and S deficiencies, and paroxysmal nocturnal hemoglobinuria are associated with IC (47).

### Illicit Drugs and Medications

Illicit drugs, mainly cocaine and methamphetamines, have been implicated in the pathogenesis of IC in otherwise healthy young subjects (27).

Elralah et al. performed a case control study during a period of 9 years comparing 19 patients with cocaine-induced IC with 78 patients with IC without cocaine use. They noted that patients with cocaine use had a younger age and a significantly higher mortality rate than the rest patients (48). Chronic use of methamphetamines also has been considered to predispose to colonic ischemia, as they are sympathomimetic drugs causing vasoconstriction and may damage the colonic mucosa (49).

Other medications have been also considered as risk factors for the development of IC, especially of the non-occlusive type, such as antihypertensives (calcium channel blockers), diuretics, estrogens, danazol, and NSAIDs (34, 46). Particularly estrogens and oral contraceptives are major predisposing factors for IC, as they provide a sequence of thromboembolic events within the mesenteric or hepatic veins, including intravascular coagulation with concomitant inhibition of the process of fibrinolysis (50). In addition, Rasmussen et al. showed that the administration of third generation of hormonal contraceptives may lead to rectosigmoid ischemia, associated with/without hematochezia (51).

### Young Patients

Kimura et al. retrospectively in five hospitals in Japan studied the characteristics of young-onset IC (13). They found that young IC patients had a higher prevalence of a smoking habit and hyperuricemia and a lower prevalence of conventional risk factors, such as hypertension and past history of abdominal surgery compared with the elderly group.

In the young age group, long-distance running is also associated with the development of occult lower gastrointestinal tract bleeding due to IC, the so-called “runner’s colitis” (24, 25, 52) These people are generating cardiac outputs of more than 25 L/min, when they incur their colitis. Splanchnic vasoconstriction, polycythemia, hypovolemia, and hyponatremia may lead to this type of IC (24, 25). This occurs mainly in 100-mile ultramarathon runners; adequate hydration, avoiding of caffeine, alcohol, NSAIDs, and high energy or hypertonic food or drink during endurance running are highly recommended for these athletes.

## Diagnostic Evaluation

Patients with IC have usually vague and atypical presentation, and in most cases, an inciting event can hardly be identified. Therefore, diagnosis of IC is often delayed while exploring more common etiologies.

Laboratory studies are usually non-specific for IC and are usually unhelpful. A complete blood count, metabolic panel, and liver function tests are required to assess the physiological status of the patient. Leucocytosis is a frequent finding and more prominent in advanced disease. Tissue injury markers, although non-specific, such as lactate, amylase, lactate dehydrogenase, and creatine kinase, should also be obtained. Abnormal findings such as elevated lactate, metabolic acidosis, and significant base deficit may occur in cases of severe ischemia, gangrene, and/or necrosis of the bowel wall and usually represent late signs (46).

### Imaging Studies

Plain abdominal X-rays are rarely helpful in the diagnosis of IC. At early stages, they may show a non-specific gas pattern in the bowel loops or ileus. Abdominal X-rays are helpful to exclude visceral perforation in the first place, as this requires emergency treatment.

As the disease progresses, submucosal hemorrhage or edema may result in focal mucosal thickening, the widely known “thumbprinting.” *Pneumatosis intestinalis* may also occur if mucosal damage has taken place with passage of gas into the bowel wall; this condition is considered by many diagnostic for IC (1, 46). However, all above signs are more evident on computerized tomography.

#### Computerized Tomography

**Figure 1:**
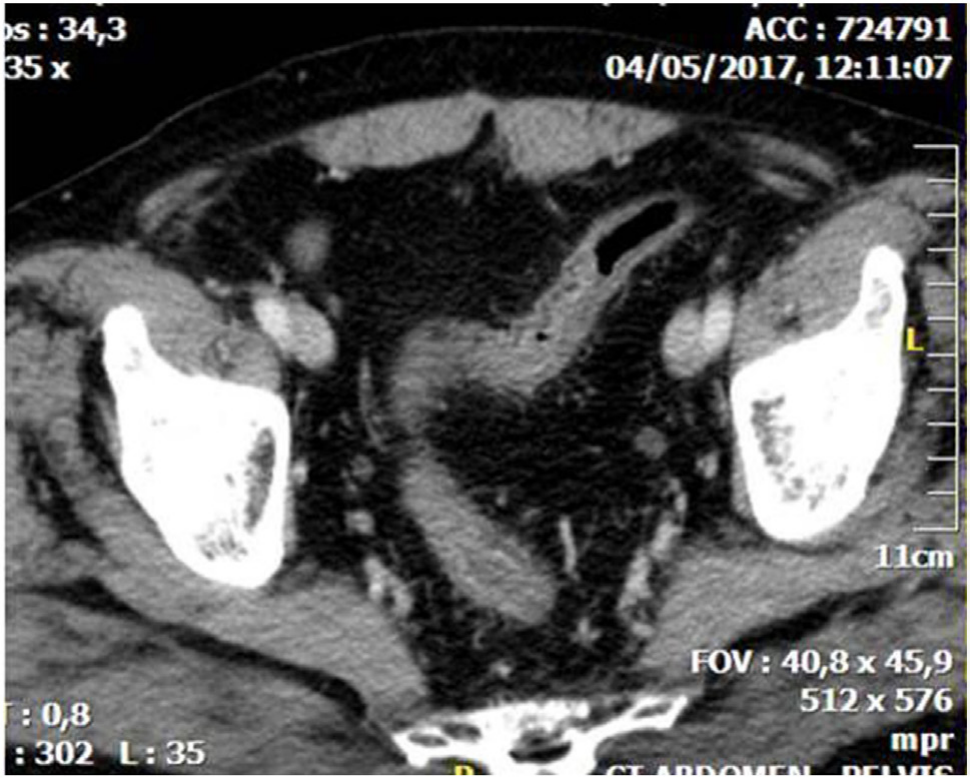
Abdominal CT showing diffuse thickening with layering of the wall of the sigmoid due to whole thickness ischemia.

**Figure 2:**
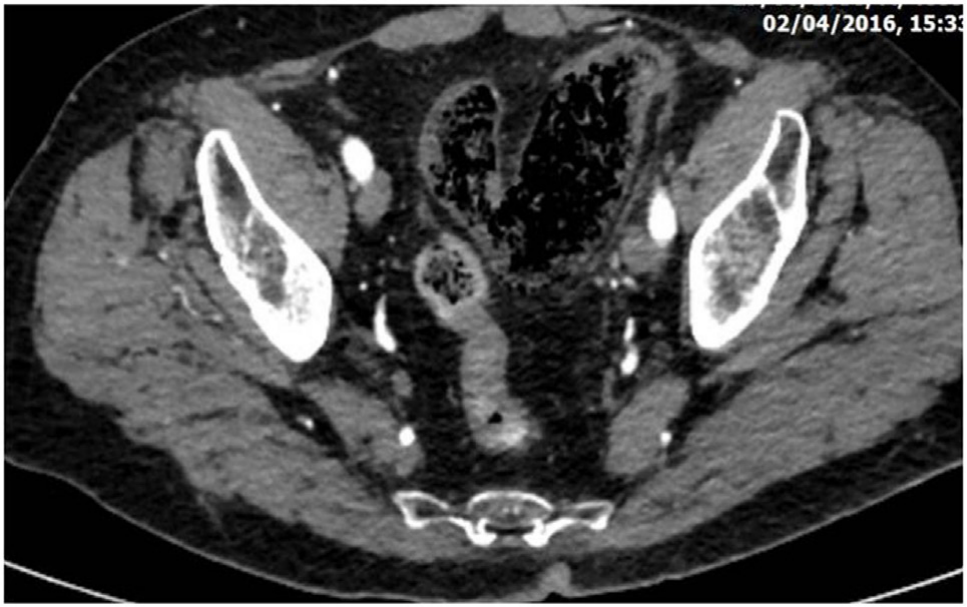
Abdominal CT revealing diffuse dilatation of the sigmoid with gas infiltration of its submucosa due to severe whole thickness ischemia (the gangrenous subtype).

**Figure 3:**
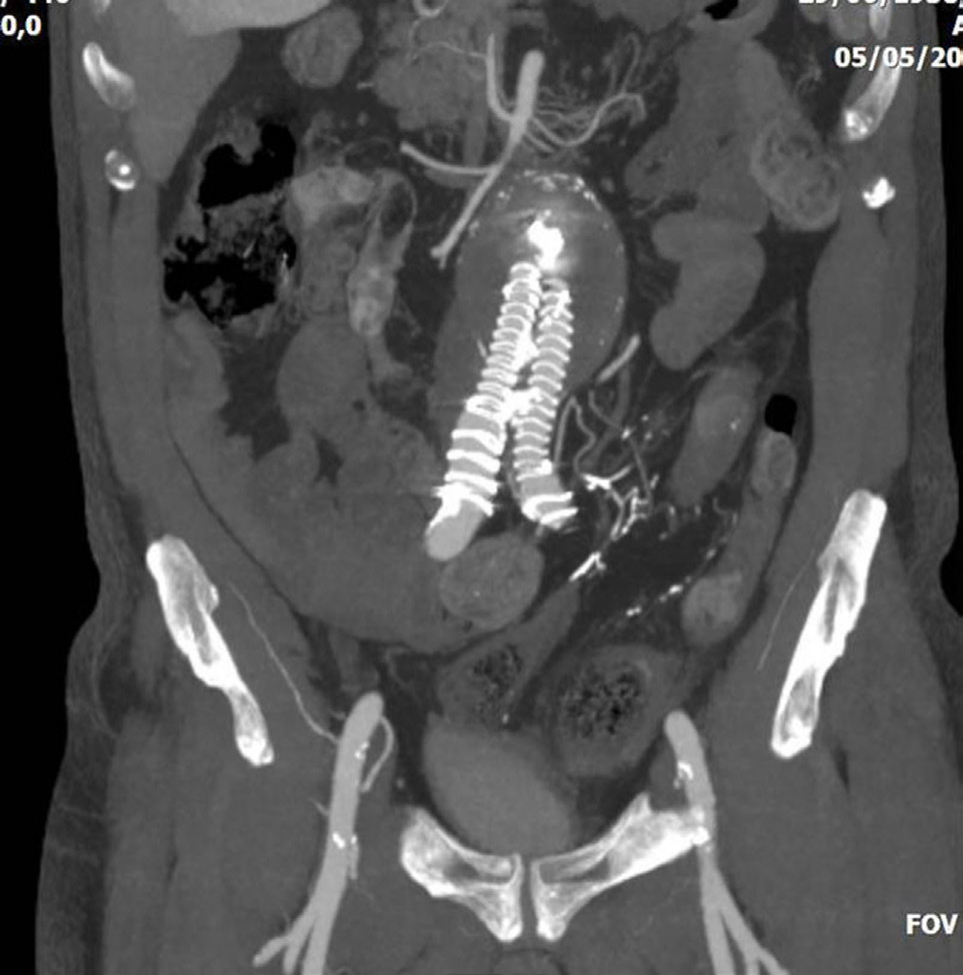
Abdominal CT of a 75-year-old patient who underwent endovascular abdominal aortic aneurysm repair with a covered aortic stent-graft. The patient had a type II endoleak *via* inferior mesenteric artery, and was treated with direct puncture and embolization with glue. Later, the patient presented severe colonic ischemia with sepsis. Embolic material is evident in the inferior mesenteric artery, whereas the sigmoid colon appears with thickened necrotic wall.

CT is the most helpful imaging examination for the patient with a suspicion of IC. It can exclude other causes of acute abdomen, identify the site, extent, and source of ischemia, and detect complications associated with severe disease. In mild forms of the disease, the CT may appear normal. In more advanced but non-gangrenous forms of the disease, the CT will often show colonic wall thickening, thumbprinting, colonic dilatation, and pericolic fat stranding with or without the presence of ascitic fluid (46). The colonic wall thickening is usually circumferential following a homogeneous or heterogeneous pattern, depending on the degree of inflammation, edema, and/or bleeding (Figures 1 and 2) (35, 53–55). The *double halo* or *target sign* may be present (54). A very common sign, segmental bowel wall thickening, is seen in most cases with an average wall thickness of 8–9 mm (55). Emboli or thrombus occluding the inferior mesenteric artery is occasionally seen, while the corresponding colonic wall appears thin due to the lack of reperfusion. The IMA occlusion is detected at the enhanced CT as defect in the lumen (56, 57) (Figure 3). Air within the mesenteric or the portal venous system or pneumatosis coli is a serious finding indicating bowel infarction (Figure 2); however, it can be present in other conditions, such as infectious colitis, COPD, steroid treatment, chemotherapy, radiation, or acquired immunodeficiency syndrome (AIDS) treatment (46, 53).

CT angiogram is often required as it offers the advantage of detecting abnormalities of the mesenteric vasculature due to atherosclerosis or thromboembolic events (35, 58). It is frequently performed following the diagnosis of IC to look for the etiology of IC. The rate of performing CT angiogram is several studies have ranged from 0 to 28.8% (35, 58). Arterial stenosis of the IMA is often encountered in CT angiograms performed for the evaluation of patients with IC (59). Although it has not presented superiority than the conventional contrast-enhanced CT scan, CT angiogram has additional disadvantages including increased exposure to radiation, potential nephrotoxicity due to contrast use, and increased costs (35). Therefore, it is reserved for severe cases especially with right colon involvement.

#### Ultrasonography

Ultrasonography, a non-invasive and readily available test, has the ability to detect mural thickening of the colon and suggests this pathology in patients with abdominal pain. Therefore, parietal thickening of a segment of the large bowel with a length of more than 10 cm, in a symptomatic patient with advanced age (>50 years), is a strong indication for IC (60). According to Lopez et al., abdominal sonography has a high positive predictive value in detecting IC (PPV 87.5%). In addition, the sensitivity of ultrasound for detecting parietal thickening in IC can reach up to 93% (61). However, ultrasonography cannot detect intestinal pneumatosis, which is detectable by the CT imaging. Moreover, CT is preferable than ultrasound in identifying cases of IC after AAA repair, since the colonic wall is often not thickened in these cases and the diagnosis is made in the absence of contrast enhancement of the diseased colon (60).

#### Endoscopy

In the absence of peritonitis, endoscopy of the lower gastrointestinal tract is the diagnostic test of choice to evaluate the degree of the mucosal ischemia. Colonoscopy should be performed on the unprepared colon within 48 h of the onset of symptoms if the CT shows atypical findings (e.g., thickened segment of colon) (14). Colonoscopy allows detection of mucosal pathology by directly visualizing the intestinal mucosa and helps to establish the diagnosis by obtaining biopsy specimens (47). After the first 48 h, the purple submucosal hemorrhages dissipate, scattered ulcers gradually develop, and sloughing occurs.

Endoscopic findings such as edematous and fragile mucosa, segmental erythema, petechial hemorrhages, scattered erosion, and longitudinal ulcerations are highly suggestive of IC (1).

Hemorrhagic nodules visualized at colonoscopy represent submucosal bleeding and are equivalent to thumbprints on barium enema studies. Segmental distribution of these findings is suggestive of IC (47). An endoscopic finding, the so-called *colon singlestripe sign* represents a single line of erythema with erosion or ulceration placed along the longitudinal axis of the large bowel. This sign is highly suggestive of ischemic injury and indicates a milder course than a circumferential ulcer (47). More severe findings include loss of haustral markings, cyanosis, and gangrene (necrosis) of the colonic wall.

The pathological findings from biopsies are often non-specific. They include erosion, granulation tissue hyperplasia, bleeding in the lamina propria, and macrophages with hemosiderin pigmentation in the submucosa (1, 62). Advanced ischemia will reveal loss of the epithelium, with the presence of inflammatory cells and congestion in the submucosa (46).

In the acute phase, colonoscopy should be performed with minimal insufflation to avoid excessive distention of the colon, which could worsen the existing ischemia of the colonic wall. CO_2_ insufflation is preferable, as CO_2_ is rapidly absorbed and exerts a vasodilating action. Bowel preparation prior to colonoscopy is not indicated, as this may induce toxic dilation or perforation of the colon.

### Treatment

#### Conservative

The treatment of a particular type of IC depends upon the severity of the disease and requires a specific plan of treatment. Clinical patterns vary from mild transient colitis to fulminant ischemia with gangrene of the colonic wall.

The transient form of IC is usually managed conservatively and has a relatively good prognosis (Table 2) (50). As the underlying cause of IC is hypoperfusion, it is mandatory to optimize perfusion of the ischemic region by administering intravenous fluids for resuscitation, optimizing cardiac output, and using supplemental oxygen (46). Any vasoconstrictive agents (vasopressors, digitalis) should be discontinued, and the patient should be placed on bowel rest; if there are signs of ileus, a nasogastric tube should be placed. The patient should be given *nil per os* at the beginning, followed by liquid diet or total parenteral nutrition to reduce intestinal oxygen requirements (15, 63).

**Table 2:** Algorithm for the management of ischemic colitis (IC).

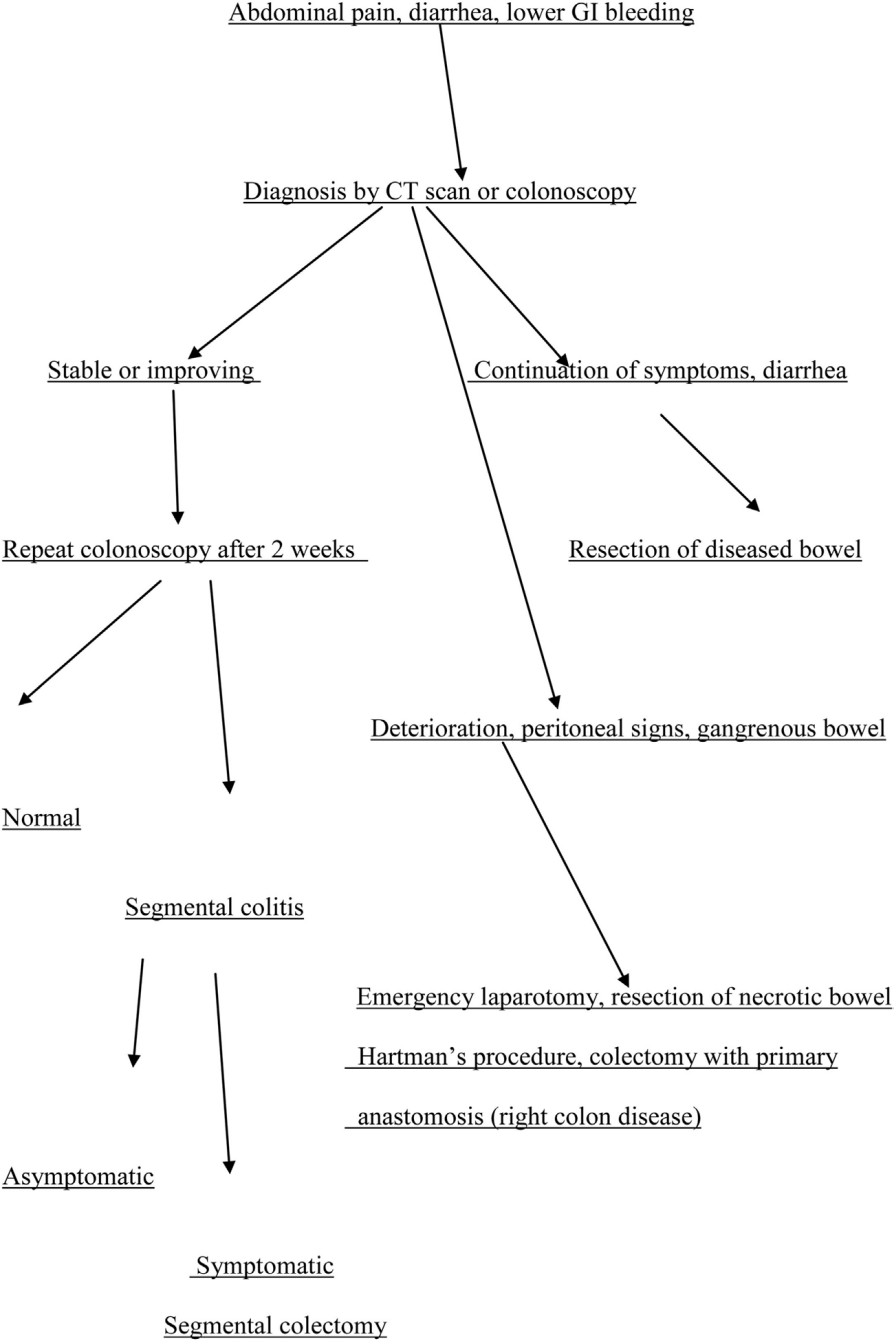

The use of broad spectrum antibiotics to cover both aerobic and anaerobic coliform bacteria is primarily recommended, as the disease process can cause disruption of the intestinal mucosal barrier and may lead to bacterial translocation to the patient’s circulation (7). A third-generation cephalosporin in combination with metronidazole is usually required to reduce the process (15, 50). Repeat physical examination, continuous patient monitoring of the total blood cell count, electrolyte levels, and serum lactate are indicated until the patient’s condition improves.

#### Surgical

In cases with no clinical improvement within 24–48 h (moderate disease), repeat colonoscopy or imaging of the mesenteric vasculature with CT angiography is necessary to reevaluate the severity and degree of the disease (46). In addition, consultation with the surgical service is required (63). Increasing abdominal tenderness with guarding and rebound tenderness, fever, uncontrollable bleeding, and paralytic ileus indicate possible infarction of the colon (severe disease) and require urgent laparotomy and removal of the necrotic part of the colon (Table 2) (47). Interestingly, in this gangrenous form, there is absence of diarrhea and hematochezia, and hypoalbuminemia (36). The decision to operate can be helped with the use of laparoscopy. The latter can detect the presence of transmural gangrene of the affected part of the large bowel, with/without perforation and peritonitis (64). Bed side colonoscopy and diagnostic laparoscopy are useful options especially for critically ill patients in the ICU, when there is suspicion for IC (65).

Colon resection is required in case of gangrenous bowel and is usually performed with open laparotomy. The extent of bowel resection can be scheduled preoperatively based on CT imaging or colonoscopy. Colonoscopy is more useful to determine the degree and extent of severe ischemia, as this is usually limited to the mucosa and the submucosa (46).

**Figure 4:**
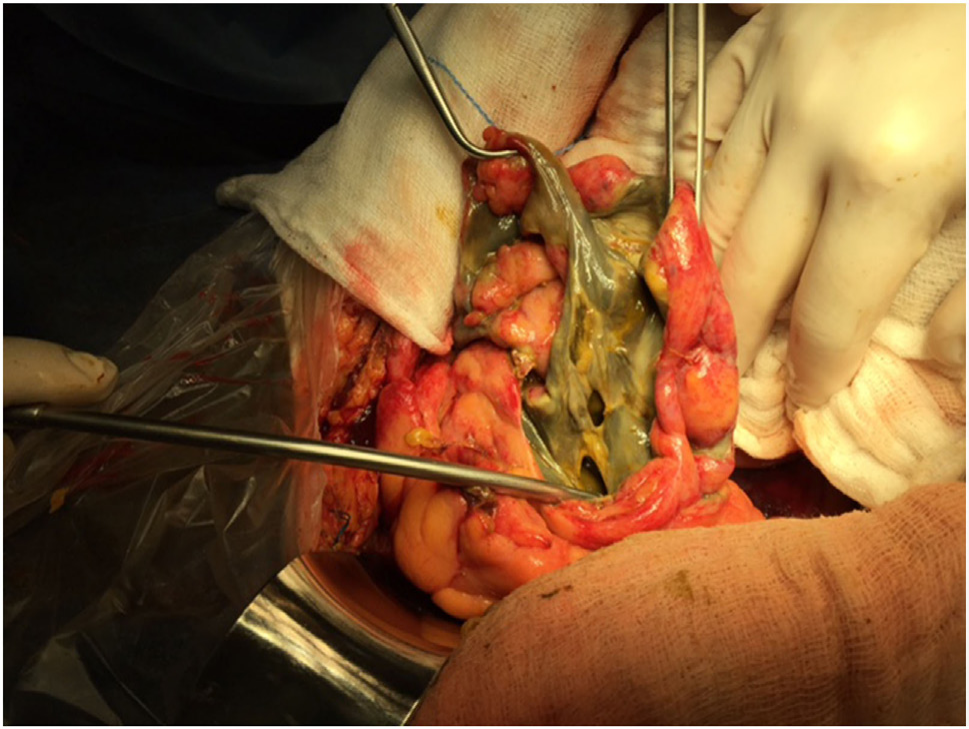
Colon infarction due to occlusion of the IMA may have devastating results. In this case there is gangrene of part of the sigmoid colon, which caused extensive rupture of the colonic wall and feculent peritonitis. Hartmann’s procedure was performed in this case with positive result.

The decision for anastomosis depends on the patient’s general condition. For patients with a trend for ongoing ischemia, there is a need for additional colon resection in up to 25% of these patients (66). Therefore, it is preferable to leave the bowel in discontinuity and return for a second laparotomy (46). Right hemicolectomy with ileostomy is usually performed for right-sided colonic ischemia and necrosis. However, in a stable patient with an uncomplicated right colon ischemia without any sign of perforation or peritonitis, resection with primary anastomosis is advised. Left-sided colonic ischemia is managed with a Hartmann’s procedure, in case of necrosis of the sigmoid and the descending colon (Figure 4; Table 2). More extensive ischemia in the large bowel with massive bleeding may urge for subtotal colectomy and end ileostomy. Reanastomosis and ostomy closure are usually done after a period of 4–6 weeks (15).

The above procedures may be done also in cases of chronic ischemia, i.e., chronic segmental colitis with recurrent sepsis, and colonic strictures that cause obstructive symptoms. In patients who have developed a stricture after an acute episode of IC, or have a stricture as a result from chronic ischemia, segmental colectomy is indicated. Moreover, chronic colitis resulting from continuous colon ischemia or unhealed areas of ischemic mucosa, should be treated with elective colectomy (46, 67). As an alternative to surgery, endoscopic dilatation or stenting of short strictures can be used, but the results have not been studied well yet.

Laparoscopic second-look procedure is preferable than surgical second-look. Reoperation should be performed within 24 to 72 h postoperatively. A small delay in second-look operation (72 h) promotes the viability of the colonic mucosa and the anastomotic healing. This procedure offers a survival rate of almost 65% (68).

### Prognosis

Prognosis of IC depends upon the location and extent of the disease, coexistent diseases, and whether or not the patient’s condition requires emergency surgery (46, 69). The severity of the IC and the overall mortality is higher in right-sided disease (70). The overall mortality is about 22% (71).

Sun et al. performed a systematic review in 2823 patients with IC from 22 studies (72). The prognostic predictors for surgery or mortality, which were most frequently reported included right-sided IC, peritonitis, shock or arterial hypotension (<90 mmHg), tachycardia, male gender and lack of rectal bleeding. In this study, right-sided IC occurred in 277 cases, with an incidence of adverse outcomes of 48.4%, while in the non-right colonic involvement group, the incidence was significantly lower at 12.1%.

In a few reports, IC may have an isolated form, with limited extension of the ischemic area in the colon (14, 20). Although this form affects a limited part of the large bowel, it was unexpectedly shown that it is associated with unfavorable outcomes compared with to non-isolated right IC (19, 73).

The evolution of the disease depends largely on the degree of ischemic damage in the colonic wall. The majority of patients respond well to conservative treatment; these patients will have improvement in their symptoms within 2–3 days and complete clinical recovery within 2 weeks, as mucosal regeneration and healing occur during that period (46). The rest 20% of patients who will eventually require emergency surgery have a mortality rate of 10–75%, despite aggressive medical or surgical treatment (36, 74). High mortality is due to the associated comorbid conditions, such as ischemic heart disease, cerebrovascular disease, and peripheral vascular disease. However, other authors report a moderate overall operative mortality (38%), as is the case of a population-based study from Iceland (2). If the original surgery involved aortic grafting, there is also a high risk of subsequent graft infection (59).

Segmental colectomy is followed by the creation of a proximal ileostomy or colostomy. In Longo’s series (75), 75% of patients, requiring segmental colon resections with stomas, will have their stomas closed. However, in patients with IC of the whole colon, only one-third will eventually have their colostomies closed.

The development of strictures is expectable in patients with severe transient colitis. Endoscopy should be performed at regular 3–4 months intervals to assess the stricture condition and allow mechanical dilatation. However, elective surgical resection with primary anastomosis may be finally required.

In previous studies, the following poor prognostic factors have been found: old age, hemodynamic instability at an early stage of IC, persistent colonic paralysis, right colon disease, hypertension, and renal insufficiency with hemodialysis, malignant tumors (46, 76). End-stage renal disease predisposes to severe IC because of continuous thrombi generation due to dialysis and repetitive hemodynamic causes. Moreover, in hemodialysis patients, rapid exchange of body fluids and the presence of hypotension may cause contraction of the mesenteric arteries, especially the superior one, thereby inducing IC of the right colon (4).

Sadler et al. in a population-based US study showed that patients requiring surgery have a higher mortality rate. Also patients of lower socioeconomic status have a worse prognosis, probably because they also have cardiovascular risk factors (e.g., smoking). They also found that severe co-morbidities, such as liver disease, renal disease, and congestive heart failure, usually increase mortality (Table 1) (37).

Noh et al. performed a retrospective analysis of 50 patients who were operated on for acute non-occlusive IC (21). They found that postoperative mortality was associated with coronary artery disease, preoperative nephropathy, previous history of cardiovascular surgery, ASA score ≥4, surgical delay ≥3 days, preoperative hemodynamic instability, and use of adrenergic vasopressors. In addition, a preceding cardiovascular operation and surgical delay ≥3 days were independent risk factors for postoperative mortality.

The long-term prognosis for this disease is acceptable after meticulous management and follow-up of these patients. Cosme et al. studied retrospectively 135 patients with IC (77). They found recurrence rates of 2.9 and 9.7% at 1 and 5 years, respectively. Five-year survival rate was 69%, but cause of death was unrelated to IC in most cases.

## Conclusion

The vague symptomatology and atypical physical findings make diagnosis of IC difficult, even in the hands of experienced clinicians. The presence of related risk factors can help in early detection of this severe disease. The majority of cases can be managed non-operatively and usually have good prognosis. In severe cases, when colon necrosis is anticipated, early surgical intervention is required and is usually associated with increased morbidity and mortality rates.

## Author Contributions

EM and DT: study design and data, paper writing. TK, IL, and DS: study data and references. GS and KM: vascular Surgery section. CK: radiology section. AM: study supervisor.

## Conflict of Interest Statement

The authors declare that the research was conducted in the absence of any commercial or financial relationships that could be construed as a potential conflict of interest.
